# Multi-Loculated Catamenial Pneumothorax: A Rare Complication of Thoracic Endometriosis

**DOI:** 10.7759/cureus.17583

**Published:** 2021-08-30

**Authors:** Grace Staring, Fátima Monteiro, Ivone Barracha, Rosa Amorim

**Affiliations:** 1 Internal Medicine, Centro Hospitalar do Oeste - Unidade de Torres Vedras, Torres Vedras, PRT; 2 Internal Medicine, Centro Hospitalar do Oeste - Unidade Caldas da Rainha, Caldas da Rainha, PRT

**Keywords:** catamenial pneumothorax, endometriosis, hormone therapy, total hysterectomy, video-assisted thoracoscopic surgery (vats), deep infiltrating endometriosis (die)

## Abstract

The presence of endometrial tissue outside the uterine cavity is known as endometriosis. Catamenial pneumothorax (CP) is a recurrent spontaneous pneumothorax that occurs in women of childbearing age. Thoracic endometriosis is a rare clinical entity, and CP is the most common presentation. Imaging diagnosis is based on computed tomography (CT) scans and magnetic resonance imaging (MRI), detecting blood products in endometrial deposits. We report a case of right CP in a 37-year-old woman with chest pain and dyspnea 48 hours after the onset of menstruation. The pneumothorax was drained, continuous hormonal therapy was started, and she underwent video-assisted thoracoscopic surgery (VATS), which revealed multiple diaphragmatic fenestrations and a solitary nodular thickening in the diaphragmatic pleura (endometrial deposit). After pleurodesis, multiple CP recurred, and later underwent a total hysterectomy. CP is the most common form of thoracic endometriosis and should be suspected in women of childbearing age.

## Introduction

The presence of endometrial tissue outside the uterine cavity is known as endometriosis and affects approximately 5%-10% of women of childbearing age. Although this ectopic tissue (stroma and small glands) is more common at the pelvic level - ovaries, uterosacral ligaments, pouch of Douglas and broad ligaments - it can occasionally arise outside the pelvic cavity (in the brain, skin, chest or abdomen). Thoracic endometriosis is a rare clinical entity [1,2] and catamenial pneumothorax (CP) is the most common manifestation, representing approximately 80% of cases [3]. The etiopathogenesis is not clear, but the most recognized theory describes the retrograde implantation of endometrial tissue by lymphatic and/or hematogenous dissemination or by the presence of congenital diaphragmatic defects.

## Case presentation

**Figure 1:**
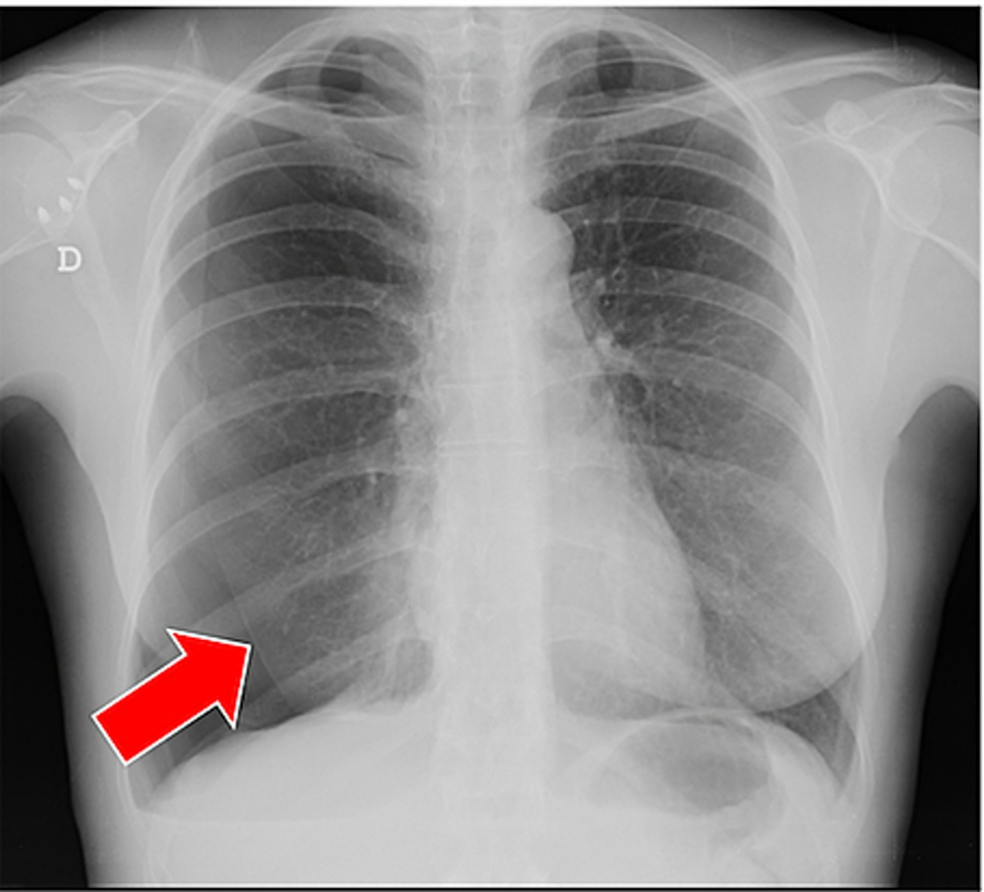
Upright posterior–anterior chest radiograph Upright posterior–anterior chest radiograph revealing a right spontaneous pneumothorax during menses (*katamênios* in greek). The subtle extrapulmonary increased lucency of the pneumothorax is outlined by the visceral pleura, a sharp thin radiopaque linear shadow (red arrow). The lung is partially collapsed with no lung markings beyond the visceral pleura.

**Figure 2:**
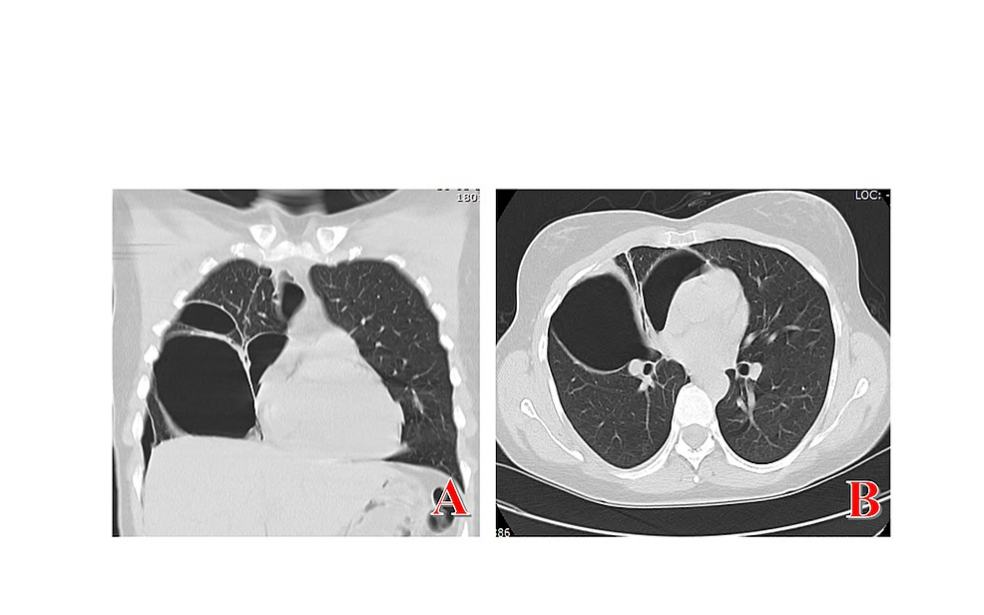
Chest computed tomographic (CT) scans Multiloculated pneumothorax on chest CT scans (coronal section A, axial section B). CT scan shows multiple pockets of loculated gas in the right hemithorax due to multiple adhesions from previous recurring bouts of pneumothorax.

We report the case of a non-smoker 37-year-old woman, admitted to the Emergency Room (ER) with chest pain, moderate dyspnea and irritating cough with 48 hours of evolution. The symptoms started days before menstruation and the duration was about four to five days, coinciding with the duration of the hemorrhagic period, decreasing or disappearing with the end of the menstruation. She reported other similar episodes of gradual and repetitive worsening during menstruation, starting in adolescence. Two years before the first observation in our ER, the patient underwent computed tomography (CT) scan of the chest, reported as normal. She denied weight loss or other relevant medical findings. Regarding the gynecological history, there was a pregnancy with a eutocic birth six years previous, and no history of documented pelvic endometriosis. Physical examination revealed decreased breath sounds in the right hemithorax. Blood analysis was unaltered and chest x-ray (Figure 1) and CT scan revealed right pneumothorax, prompting a three-day hospital stay in the internal medicine ward for chest drainage. Despite the absence of clinical and imaging evidence of pelvic endometriosis, the hypothesis of CP was taken into account. She started first-line treatment with hormonal agents, a continuous contraceptive pill (drospirenone/ethinylestradiol), with remission of respiratory symptoms. However, three months after this initial episode, the patient presented a recurrence of the condition, this time with tomographic evidence of a multiloculated pneumothorax on the right (Figure 2). She was transferred to the cardiothoracic surgery department, where she underwent video-assisted thoracoscopic surgery (VATS) with an observation of multiple diaphragmatic fenestrations and nodular thickening of the diaphragmatic pleura collected for histology. The final histological result of the surgical specimen confirmed endometrial tissue, which led to the conclusion of CP related to endometriosis. Two weeks after surgery, she repeated the right pneumothorax and underwent a new VATS with pleurodesis. Shortly after the last surgical procedure, the gynecology colleague changed the hormone therapy initially instituted to goserelin, a gonadotropin-releasing hormone agonist (GnRH agonist) and dienogest, a progestogen-only pill (POP) for three months. Six months after surgery and hormonal therapy, she presented a recurrence with small right pneumothorax, which resolved with rest, and treatment failure was declared. She then underwent a total hysterectomy 11 months ago and is in precocious menopause, with no reported complications.

## Discussion

CP is rare. It occurs during the menstrual period and is due to lung collapse. It is considered secondary to endometriosis in the pleural cavity and causes one-third of spontaneous pneumothoraces in women of childbearing age [1,2]. Although there has been great progress in imaging techniques and minimally invasive procedures, the lack of macroscopic findings in surgery makes the clinical syndrome still underdiagnosed with a reported incidence of only 3% to 5% [1-6]. This clinical entity occurs more frequently in women between 30 and 50 years, who usually have a history of infertility, severe endometriosis and recurrent spontaneous pneumothorax within the 72 hours preceding the onset of menstruation [5,6]. Almost 5%-10% of women of childbearing age are affected by endometriosis, which can be extrapelvic. There are two types of thoracic endometriosis: pleural and pulmonary endometriosis. The first is characterized by chest pain associated with CP, hemothorax or pneumomediastinum; the pulmonary form presents as pulmonary nodules and catamenial hemoptysis [7,8]. There are three theories that explain the etiology of CP: metastatic, anatomical and hormonal. The metastatic theory assumes that transdiaphragmatic passage of endometrial tissue occurs by lymphohematogenous dissemination or by congenital diaphragmatic fenestrations. The anatomical theory requires, during menstruation, the disintegration of the cervical mucus plug, with a transfer of cells through the vagina, uterus and fallopian tubes to the peritoneum, reaching the chest through congenital fenestrations of the diaphragm. The hormonal theory determines that, during ovulation, ischemic injury and alveolar rupture develop due to pulmonary vasospasm mediated by prostaglandin F2 [5-8]. CP (associated with pleural lesions) appears almost exclusively on the right side, apparently because there is more considerable diaphragmatic lymphatic drainage on the right and by the clockwise peritoneal circulation that drags the endometrial implants to the right diaphragm [9]. The diagnostic imaging is based on CT scans to detect endometrial deposits in the lung and pleura and pelvic ultrasound, as there is usually coexistence of pulmonary and pelvic endometriosis in ~80% of cases. Magnetic resonance imaging (MRI) is considered superior to CT as it can show blood products in endometrial deposits. The combination of surgical treatment followed by a period of about six months of hormonal therapy can reach up to 45 months of follow-up without recurrence and is currently the preferred approach for treatment and prevention [10,11]. In our clinical case, we found multiple diaphragmatic fenestrations associated with nodular thickening/endometrial implant on the right posterior diaphragm surface. The lesion was removed followed by chemical pleurodesis associated with hormonal treatment, but without success [10-12]. A total hysterectomy was performed, and the patient has been in menopause for 11 months, stable. The anatomopathological report of the uterus and appendages did not reveal evidence of endometriosis, our patient being the first case of non-gynecological deep infiltrating endometriosis.

## Conclusions

In young women with pneumothorax during menstruation, CP should always be suspected. Delay in treatment usually leads to recurrence and worsening. It is necessary to carefully observe and remove the pleural lesions (both parietal and visceral) and surgically repair the diaphragmatic fenestrations. The start of hormonal therapy is also recommended, as it seems to help maintain the results achieved with surgery. Rarely, due to the failure of less invasive therapeutic strategies, total hysterectomy is the only solution to resolve the clinical condition. It is important to highlight the importance of a multidisciplinary team - internal medicine, pulmonology, cardiothoracic surgery and gynecology - in conducting this case, each case, in search of success.
